# N6-methyladenosine demethylase FTO targets pre-mRNAs and regulates alternative splicing and 3′-end processing

**DOI:** 10.1093/nar/gkx778

**Published:** 2017-08-30

**Authors:** Marek Bartosovic, Helena Covelo Molares, Pavlina Gregorova, Dominika Hrossova, Grzegorz Kudla, Stepanka Vanacova

**Affiliations:** 1 CEITEC–Central European Institute of Technology, Masaryk University, Brno 62500, Czech Republic; 2 MRC Human Genetics Unit MRC IGMM, University of Edinburgh Western General Hospital, Crewe Road, Edinburgh EH4 2XU, UK

## Abstract

N6-methyladenosine (m^6^A) is the most abundant base modification found in messenger RNAs (mRNAs). The discovery of FTO as the first m^6^A mRNA demethylase established the concept of reversible RNA modification. Here, we present a comprehensive transcriptome-wide analysis of RNA demethylation and uncover FTO as a potent regulator of nuclear mRNA processing events such as alternative splicing and 3΄ end mRNA processing. We show that FTO binds preferentially to pre-mRNAs in intronic regions, in the proximity of alternatively spliced (AS) exons and poly(A) sites. FTO knockout (KO) results in substantial changes in pre-mRNA splicing with prevalence of exon skipping events. The alternative splicing effects of FTO KO anti-correlate with METTL3 knockdown suggesting the involvement of m^6^A. Besides, deletion of intronic region that contains m^6^A-linked DRACH motifs partially rescues the FTO KO phenotype in a reporter system. All together, we demonstrate that the splicing effects of FTO are dependent on the catalytic activity *in vivo* and are mediated by m^6^A. Our results reveal for the first time the dynamic connection between FTO RNA binding and demethylation activity that influences several mRNA processing events.

## INTRODUCTION

Reversible N6-methyladenosine (m^6^A) is the most prevalent modification in eukaryotic mRNA (1–8). It was initially discovered together with the 7-methylguanosine cap already in the 1970s (9–16), although the precise locations of m^6^A within mRNAs were uncovered by transcriptome-wide studies only in 2012 (1,2). The most striking feature that emerged from these studies was the widespread presence of m^6^A in more than half of all mammalian mRNAs and few hundred non-coding RNAs (ncRNAs) (1,2,17). The RNA modification by m^6^A affects multiple aspects of mRNA metabolism, such as alternative splicing (AS) (18–21), stability (3–5,8,22), translation efficiency (23–26) or localization (27).

The dynamics of m^6^A is coordinated by an interplay between the m^6^A writers, erasers and readers that deposit, remove or specifically bind m^6^As, respectively. The mRNA m^6^A writer is represented by a multi-subunit methyltransferase complex consisting of the core MMW complex that is formed by the methyltransferase-like 3 and 14 (METTL3 and METTL14), Wilms' tumor 1-associating protein (WTAP) and the protein Virilizer homolog (KIAA1429) (5–7). METTL3 possesses methyltransferase activity, whereas the METTL14 is catalytically inactive and serves as an RNA binding platform for METTL3, enhancing its activity (7,28,29). WTAP and KIAA1429 are auxiliary proteins, which promote RNA methylation *in vivo*, although the exact mechanism is still unknown (5–7). Most of the m^6^A marks are found within the consensus motif DRACH (D = A, G or U; R = A or G; H = A, U or C) (7,30). Whereas DRACH occurs rather frequently in mRNA, only a fraction of the sites are methylated *in vivo*. Notably, m^6^A methylation is non-randomly distributed throughout the mRNA body being enriched particularly around the transcription start site (TSS) and the stop codon/beginning of the last exon (1,2). However, the cause of the enrichment in such specific sites in mRNAs is currently unknown. The m^6^A is interpreted by m^6^A-binding proteins - readers. Direct m^6^A readers possess the RNA-binding YTH domain, which is responsible for m^6^A recognition (2,4,6) and mediates divergent roles of m^6^A in the RNA metabolism (4,18,25). The best characterized is the role of YTHDF2, which negatively regulates stability of m^6^A modified mRNA (4). This function of m^6^A has a particularly important role in the regulation of pluripotency in embryonic stem cells (3,8).

The presence of m^6^A can change structural properties of the adjacent RNA region and influence binding of RNA-binding factors (20). To date, this feature appears to play a role in the regulation of alternative splicing (20). Other studies linked m^6^A to a transcription factor activity (31) or regulation of alternative polyadenylation (poly(A)) site usage (32) underscoring the role of m^6^A in coupling transcription with pre-mRNA processing. However underlying molecular mechanisms are not well understood (32).

Adenosine methylation can be directly reversed by so called erasers, represented in mammals by at least two m^6^A-specific demethylases—ALKBH5 (AlkB homolog 5) and FTO (fat mass and obesity-associated) (27,33). They belong to a broader family of AlkB dioxygenases. ALKBH5 localizes to the nucleus and its depletion leads to global reduction of poly(A) RNAs in this cellular compartment, suggesting a role in the regulation of nucleo-cytoplasmic shuttling of mRNA (34). FTO is localized in the nucleus and cytoplasm (33,35) and it was linked to multiple processes including splicing, sensing of amino acids and differentiation of preadipocytes (19,36,37). Interestingly, neither ALKBH5 nor FTO display a preference for DRACH m^6^A motif *in vitro* (38). Currently, the target repertoire of ALKBH5 and FTO remains largely unknown and it is unclear to what extent they are redundant in function. The expression pattern in mammalian tissues indicates that they have distinct roles in animal physiology. ALKBH5 is highly expressed in testis and is crucial during spermatogenesis (27), whereas FTO is widely expressed in all adult and fetal tissues with the highest expression in the brain (39). Although FTO mutations were initially associated with obesity (39), this role is currently under debate (40,41). Despite the recent enormous interest in m^6^A RNA modifications the demethylases remain the least-characterized parts of this pathway.

Here, we use a combination of CLIP-seq and RNA-seq to show that FTO is a potent regulator of multiple mRNA processing events. We uncover a novel role of FTO in alternative splicing and demonstrate that FTO binding to regions proximal to alternative exons triggers their inclusion into mRNA. In addition, FTO depletion results in upregulation of terminal mRNA exons. This links FTO to the previously suggested role of m^6^A in regulation of alternative poly(A) site usage and 3′ end mRNA processing (32). Taken together, our results reveal the direct role of the FTO demethylase in regulation of several nuclear pre-mRNA processing events.

## MATERIALS AND METHODS

### Cell culture and manipulation

Human HEK293 Flp-In T-REx cell line (293T) (Invitrogen) cells were cultured in Dulbecco's Modified Eagle Medium (DMEM) supplemented with 10% fetal bovine serum (FBS) at 37°C in the presence of 5% CO_2_. Transfections of plasmids were performed using TURBOFECT reagent (Fermentas) at 70% confluency according to manufacturer's instructions.

### Knockout of FTO

Both alleles of *FTO* gene were knocked out using the nickase CRISPR–Cas9 system (42,43) according to (44). Shortly, oligonucleotide sequences targeting both strands of exon 2 of FTO were cloned into pX461 vector (Addgene # 48140) and cotransfected into the 293T cell line. Cells were checked for GFP expression after 24 h, counted and seeded with the density 0.5 cell/well in 96-well plates. Expression of FTO was analyzed in five weeks by western blot with rabbit polyclonal antibodies against FTO (Abcam, EPR6894).

### Site-directed mutagenesis

H231A, D233A double mutant of FTO was generated by site directed mutagenesis. Shortly, full length FTO subcloned in pcDNA5/FRT/TO was subjected to inverse PCR with oligonucleotide primers containing desired mutations (FTO_HDmut_fw and FTO_HDmut_rev). The PCR reaction was then digested with DpnI (NEB) to remove template DNA and transformed into *Escherichi coli* strain TOP10. Colonies were screened for the mutation and successful clones were confirmed by sequencing.

### Cloning and stable cell lines preparation

The full length FTO gene (AA 1–505) was amplified from the cDNA library prepared from 293T cells. PCR product was digested with NotI and XhoI and ligated into pcDNA5/FRT/TO plasmid (Invitrogen) containing an N-terminal FLAG tag. Successful clones were verified by sequencing.

The 293T cells were grown to 70% confluency and cotransfected with 0.6 μg of pcDNA5/FRT/TO-3xN-FLAG-FTO and 6 μg of pOG44 (Invitrogen). After 24 h, cells were transferred to 150 mm dish and stable recombinants were selected using 100 μg/ml of hygromycin B. Cells were cultivated until individual colonies were visible on the plate. Several colonies were tested for Zeocin sensitivity and doxycycline-inducible expression of FLAG-tagged protein by western blot.

### Immunofluorescence

Cells were grown on polyethyleneimine coated coverslips, washed with pre-warmed PBS and fixed in 3.7% paraformaldehyde. Fixed cells were washed with PBS, permeabilized by 0.2% TritonX-100 in PBS for 20 min and blocked in 5% horse serum in PBS. After 1 h blocking at RT, cells were incubated with anti-FTO primary antibody (Abcam, 1:250) in 3% horse serum for 1 h at RT. Cells were washed three times for 10 min with PBS, incubated with mix of Alexa488 secondary antibodies (Invitrogen, 1:800) and DAPI (Sigma, 1:500) in PBS for 30 min at RT in the dark. Cells were washed with PBST and PBS and finally sterile ddH_2_O and fixed on slides in Mowiol 4-88 (Sigma) with DABCO. Samples were imaged by Olympus FluoView-500 confocal imaging system combined with an inverted Olympus IX-81 microscope. The images were recorded using an Olympus DP70 CCD camera. All pictures were taken at 600-fold magnification. Pictures consist of 10 overlapped individual sections, which are 0.4 μm apart from each other. All pictures were processed by software FluoView (Olympus).

### CLIP-seq protocol

CLIP-seq was performed with modifications according to (45,46). Briefly, RNAse I was used for RNA fragmentation, pre-adenylated 3′ linker and truncated RNA ligase 2 (NEB) was used for on-beads 3′ linker ligation and cDNA library was amplified by 25 PCR cycles (Detailed CLIP-seq procedure can be found in supplementary methods).

### RNA immunoprecipitation

Cells expressing FLAG-tagged FTO were grown to 90% confluence, washed with ice cold PBS and lysed in lysis buffer (LB) containing 150 mM NaCl, 50 mM Tris pH 7.6, 1% Triton X-100, EDTA-free Complete Protease Inhibitor Cocktail (Roche), 1 mM DTT, RNase In (Promega). Lysates were sonicated 6 × 10 s at 7% amplitude, incubated with Turbo DNase (Fermentas) for 15 min at 37°C and cleared by centrifugation. Supernatants were subsequently pre-cleared with magnetic beads without antibodies for 1 h at 4°C. FLAG M2 Magnetic beads (Sigma) were incubated with 10 μg of yeast tRNA for 1 h at 4°C. The pre-cleared extracts were applied on the pre-blocked FLAG beads and incubated for 2 h at 4°C. Beads were washed three times with LB and the bound RNA was extracted with the TriPure reagent (Roche) according to manufacturer's instructions. The isolated RNA was treated with the Turbo DNase (Fermentas) and used as a template for cDNA synthesis by Superscript III reverse transcriptase (Invitrogen) with random hexamer priming. The cDNA was subsequently analyzed by semi-quantitative or real-time qPCR.

### DNA library preparation for RNA-seq analysis

RNA-seq was performed on a 293T 3xN-FLAG-FTO overexpressing cell line, FTO knockout cell line as well as the 293T cell line. RNA was isolated using TriPure Isolation Reagent (Roche) and ribosomal RNAs were depleted with RiboMinus Eukaryote System v2. Depletion of ribosomal RNA was checked on Agilent RNA Nano Chip. The cDNA libraries were prepared with ScriptSeq-v2 RNA-seq Library Preparation Kit (Epicentre) according to manufacturer's instructions. Quality of libraries was assessed using High Sensitivity DNA Analysis Kit (Agilent). DNA libraries were sequenced on Illumina HiSeq 2000, in the 2 × 125 bp paired-end mode by the EMBL Genomics Core Facility, Heidelberg, Germany.

### High-throughput sequencing data analysis

Raw reads were trimmed and quality control was performed using TrimGalore! (Babraham Bioinformatics) with default settings. Trimmed reads were mapped to the human genome version hg19 using TopHat2 v 2.0.14 (47). CLIP-seq reads were mapped both with and without the transcripts annotation (Illumina iGenomes v. GRCh37). Both mappings were combined and mapping positions were selected based on minimal number of mismatches. CLIP-seq reads were categorized according to priority table (1. rRNA, 2. tRNA, 3. snoRNA, 4. snRNA, 5. miRNA, 6. mRNA exon, 7. mRNA intron, 8. mRNA unknown, 9. lincRNA, 10. repeat 11. antisense mRNA, 12. unannotated).

To cluster the CLIP-seq reads we dissected the human genome to 50 bp windows and calculated RPKM for all windows in CLIP-seq and input (RNA-seq). Binding clusters were filtered and selected based on two conditions. First, we calculated enrichment of FTO CLIP RPKM in cluster over input (RNAseq) and filtered only clusters, which were enriched >2-fold. Second, to remove lowly covered regions, we selected only clusters with minimum of 10 reads coverage in all three replicates summed.

Differential gene and exon expression analysis was performed using R packages DESeq2 (48) and DEXSeq respectively according to documentation (49). Genes/exons with an adjusted *P*-value < 0.05 were considered significantly DE. Top up/downregulated exons were selected based on ordered adjusted *P*-values. Numbers of exons were acquired by custom scripts from the Ensembl annotation (GRCh37.75). MISO analysis was performed according to documentation (50). The top 200 inclusion/exclusion events were selected based on their respective Bayes factor. Splicing events with Bayes factor > 10 were considered significant. Metagene analysis was performed with CGAT tool bam2geneprofile.py (51) or custom scripts. GO analysis was performed using R package GOSeq (52). RPKM calculations in features were done using bedtools (53). CLIP-seq/RNA-seq coverage plots were calculated using coverageBed from the bedtools toolset and visualized using custom scripts in R. Gene models were generated using R package GenomeGraphs (54).

Alternative poly(A) (APA) site usage was estimated using DaPars (55). APA events with adjusted *P*-value < 0.05 were considered significant. For motif analysis, binding sites were selected based on the identification of crosslinking-induced deletions in the CLIP reads. Positions with >5 reads coverage and at least 5% of deletion frequency were selected. K-mers were counted by ZOOPS (Zero or once per transcript) counting. Significance of motif enrichment was calculated by Fisher's exact test for each replicate separately, and *P*-values were combined by Fisher's method.

### Reverse transcription and qPCR analysis

Total RNA isolated with TriPure reagent according to the manufacturer's instructions was treated with DNAse (Turbo DNAse, Fermentas) and 2μg of RNA was used for reverse transcription (RT) with random hexamers and SuperScript III enzyme (ThermoFisher scientific). Typically 2 μl of 2–10× diluted cDNA was used for PCR/qPCR reaction. qPCR was performed using FastStart Universal SYBR Green Master (Roche) on Roche LightCycler 480. Each measurement was performed in three biological and three technical replicates. Differential expression was calculated using ΔΔCT method. Data were normalized to expression of the housekeeping gene HPRT. Results are expressed as means and standard errors of the mean. *P*-values were calculated using a two-tailed Student's *t*-test.

### Splicing assay by using the minigene reporter construct

To generate the splicing minigene construct, the genomic region chr5:137 495 750–137 494 549 (synthetic GeneArt Strings purchased from Life Technologies) corresponding to the AS exon 20/21 of the BRD8 gene (according to reported isoforms NM_139199 and NM_006696, respectively) and flanking intronic regions 462 bp upstream and 695 bp downstream was inserted into the pDESTsplice vector (pDESTsplice was a gift from Stefan Stamm, Addgene plasmid #32484, (56)) using the Gibson Assembly Master Mix (New England BioLabs). The pDESRsplice-BRD8-del construct lacks the 53 bp long intronic region downstream of the AS exon, which was highly covered in the FTO-CLIPseq results (it corresponds to the genomic position chr5:137 495 164–137 495 112). In the pDESTsplice-BRD8-mut construct, this 53 bp region was replaced by sequence 5′ GAGACGCCTAAGGTTAAGTCGCCCTCGCTCGCTACTATGCGGTGTAGCTCTTC 3′, which is a sequence not found in the human genome flanked by two restriction sites.

The constructs were transiently expressed in 293T WT and FTO KO cells, briefly, 1.5 μg of the reporter construct DNA was transfected to 80% confluent 293T cells in 6-well culture plates using TURBOFECT following the manufacturer's instructions (Fermentas). The cells were harvested 24 h after transfection and total RNA was isolated with TriPure reagent according to the manufacturer's instructions. The isolated RNA was treated with DNAse (Turbo DNAse, Fermentas) and 2 μg of RNA was used for reverse transcription (RT) with random hexamers and SuperScript III enzyme (ThermoFisher scientific). To assess the splicing pattern, primers annealing to the rat insulin exons 2 and 3 were used (see the Supplementary Table 1 for the sequences). RT-PCR products were resolved on agarose gels and signals were quantified by ImageJ (57).

## RESULTS

### Characterization of FTO RNA targets by CLIP-seq

**Figure 1.**
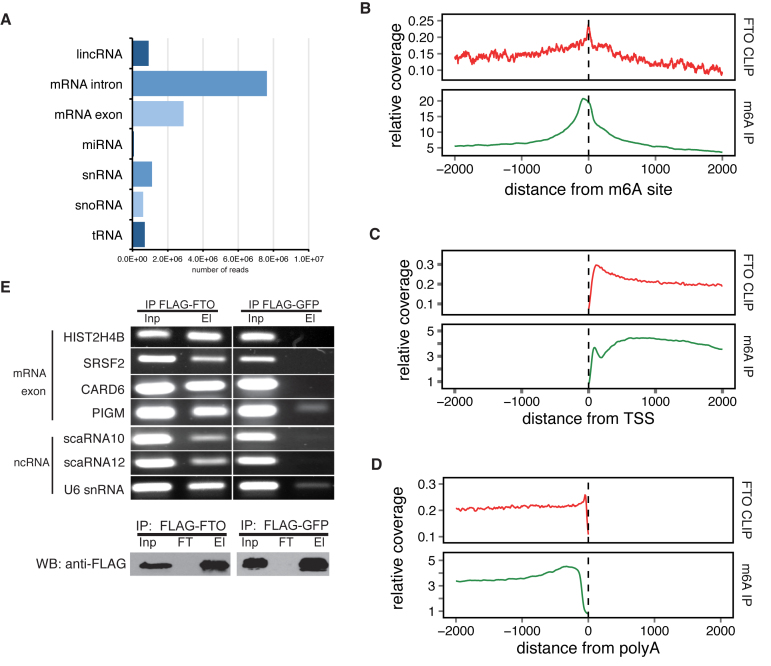
Identification of FTO targets by CLIP-seq analysis. (**A**) The distribution of CLIP-seq reads among individual RNA classes. Reads were categorized according to the priority table (See methods) (B–D) Metagene distribution of FTO CLIP and m^6^A-seq reads around positions of m^6^A (**B**), transcription start site (TSS) (**C**) and poly(A) sites (**D**). (**E**) RNA IP confirmation of FTO binding to RNAs identified by CLIP-seq (upper panel). Representative exonic and ncRNA binding sites were selected and gene names are indicated on the left. Western blot analysis of FLAG-FTO immunoprecipitation (IP) efficiency (lower panel). FLAG-GFP was used as a background control. Inp is the whole cell lysate. El is the bound fraction. FT (Flow-through) is the unbound fraction.

To characterize the RNA targets bound by FTO, we performed cross-linking and immunoprecipitation (CLIP-seq) (46) of FTO in the human embryonic kidney HEK293 Flp-In T-REx cell line (293T). To ensure high specificity of the immunoprecipitation (IP), we prepared a stable 293T cell line with inducible expression of FLAG-tagged full-length FTO and optimized the immunoprecipitation (IP) conditions and RNAse I fragmentation (Supplementary Figure S1A and B). We performed three replicates of CLIP-seq followed by high-throughput sequencing on the Illumina platform, together with three replicates of RNA sequencing of the same cell line (Input). The CLIP-seq libraries yielded 33 922 806, 40 223 089 and 96 201 502 reads, respectively. After adapter trimming and collapsing of duplicate reads, we were able to map more than 70% of the reads in all three samples to the human genome version hg19 using tophat2 (47) (762 879, 1 633 537 and 4 669 684 uniquely mapped, de-duplicated reads in three replicates). The distribution of CLIP reads in RNA classes was reproducible and most of the reads were mapped to mRNAs (42%), followed by ribosomal RNAs (rRNAs) (26%), and small nuclear RNAs (snRNAs) (4%) (Figure 1A, Supplementary Figure S1C).

To filter FTO binding sites and assess the reproducibility of the CLIP-seq datasets, we performed a read clustering analysis (see Methods for details). We used the filtering condition >10 reads in a cluster and >2-fold enrichment over input (RNAseq) in at least two replicates. This resulted in a total number of 46 186 binding clusters in 9224 exons. This is comparable to the number of peaks obtained in the recent m^6^A miCLIP analysis, which revealed 27 771 m^6^A peaks in 11,880 exons (17). We observed high correlation of read counts in FTO binding clusters as well as mRNA exons and mRNA introns (Supplementary Figure S1D). Unfiltered FTO-binding clusters with >20 reads revealed almost >90% overlap of binding clusters in at least two biological replicates (Supplementary Figure S1E). Similar percentage of overlap was obtained for other filtering conditions.

Because FTO is an m^6^A eraser, we next compared FTO binding with positions of known m^6^A modifications. Metagene analysis revealed a peak of FTO binding around known m^6^A sites (17) (Figure 1B). Moreover, we observed a 16% overlap between exons modified by m^6^A and exons bound by FTO (Supplementary Figure S2A, B).

Previous studies revealed m^6^A enrichment around transcription start sites (TSS) and stop codons (1,2). To examine whether FTO might be involved in the regulation of m^6^A abundance around these features, we compared the distribution of FTO CLIP reads in mRNA transcripts with the published m^6^A-seq results (1). Our CLIP-seq analysis revealed pronounced enrichment of FTO binding at TSS (Figure 1C) and a moderate enrichment around stop codons (Supplementary Figure S2C). Interestingly, we detected a peak of FTO enrichment at around 100nt upstream of known poly(A) sites (Figure 1D), which was different from the distribution of m^6^A reads. M^6^A was more dispersed throughout a wide region upstream of poly(A) sites (Figure 1D) indicating that it is enriched more generally throughout the 3′ UTRs and that FTO may be removing a specific subset of the marks close to 3′ends of mRNAs.

In order to look whether FTO might have a target sequence preference, we performed motif search analysis in the CLIP-seq datasets. Although, we did not observe enrichment around the DRACH motif (Supplementary Figure S2D and E), the FTO reads were enriched in several pyrimidine-rich sequences (Supplementary Figure S2E). Moreover, these motifs were reproducibly occurring in all three replicates of CLIP (Supplementary Figure S2E). However, the most enriched motif occurs only in ∼5% of FTO bound sequences (Supplementary Figure S2E). Therefore, although the newly found motifs are strongly associated with FTO binding, they do not appear essential for RNA recognition by FTO.

To validate the FTO CLIP-seq results, we performed anti-FLAG-based RNA immunoprecipitation (IP) coupled to RT-PCR (RIP-PCR) with gene-specific primers. The IP samples from FLAG-FTO cells revealed a strong signal for all RNAs examined, four mRNAs and three ncRNAs (Figure 1E). On contrary, FLAG-GFP IP, which was used as a background control, showed very weak or no coprecipitation of the tested RNAs (Figure 1E). Taken together, we provide a transcriptome-wide map of RNAs bound by the m^6^A demethylase FTO, which revealed a significant overlap with the previously identified m^6^A locations within mRNAs.

### FTO targets pre-mRNAs in the nucleus

**Figure 2.**
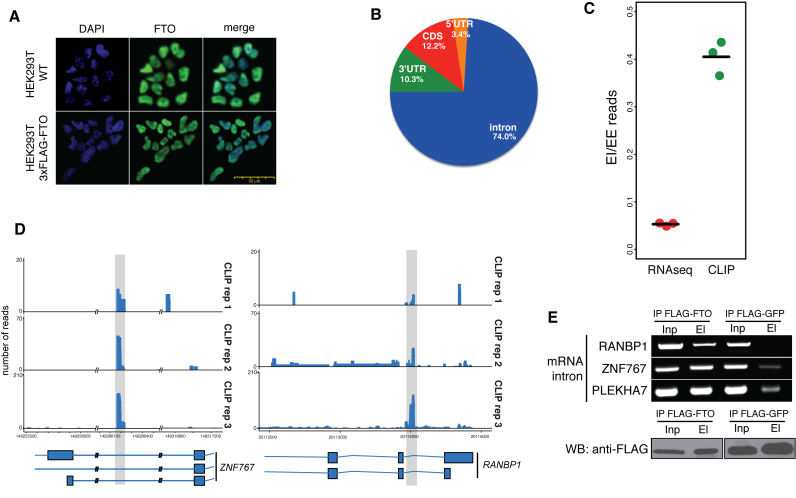
FTO binds pre-mRNAs in the nucleus. (**A**) Endogenous FTO and ectopically expressed FLAG-tagged FTO localize to the nucleus in 293T cells. Immunofluorescence detection with anti-FTO antibodies (Green). DNA is stained with DAPI (Blue) (**B**) Piechart representation of the percentage of CLIP-Seq reads corresponding to particular regions of pre-mRNAs (**C**) Comparison of the fraction of sequencing reads spanning exon-exon (EE) junctions relative to exon-intron junctions (EI) in FTO CLIP-seq and total RNA-seq. (**D**) Examples of FTO binding in intronic mRNA regions. Shaded regions highlight FTO binding sites. (**E**) RIP-PCR confirmation of FTO binding to intronic regions in genes selected based on CLIP-Seq binding sites (upper panel). Western blot (WB) analysis of FLAG-FTO immunoprecipitation (IP) (lower panel). FLAG-GFP was used as a background control. Inp is the whole cell lysate. El is the bound fraction.

To address the function of FTO binding in 293T cells, we performed immunofluorescence localization of endogenous FTO in the control 293T cells and of the 3xFLAG-FTO stably integrated in the 293T cell line. In both cases, FTO localized predominantly to the nucleoplasm (Figure 2A). Consistently with nuclear localization, the majority of FTO CLIP-seq reads that correspond to protein-coding genes fall into intronic regions (74% of all mRNA reads, Figure 2B). The remaining 26% of mRNA reads fall into 3′ UTRs (10.3%), coding regions (12.2%), and 5′ UTRs (3.4%) (Figure 2B). Moreover, the intronic binding of FTO was highly reproducible between the three replicates (Figure 2D, Supplementary Figure S1C and D).

To further examine the FTO binding to pre-mRNAs, we analyzed and compared the number of reads spanning the intron-exon boundaries and exon-exon junction regions, respectively. Our results revealed a significantly higher ratio of exon-intron spanning reads relative to exon-exon junction reads in FTO CLIP-seq compare to the total RNA input (RNA-seq) (*P* = 7.3 × 10^−5^, Student's *t*-test, Figure 2C). FTO binding to intron-containing pre-mRNA was further experimentally validated by FTO RIP-PCR. We used primers annealing and amplifying intronic regions significantly enriched in the FTO CLIP-Seq data (Figure 2D). The result in Figure 2E demonstrates that FTO coprecipitated three different intron-containing mRNAs, whereas weak or no signal was observed in samples where FLAG-GFP expressing cells were used as a background control. In summary, our experiments and analyses strongly imply, that FTO targets intronic regions in pre-mRNAs.

### Depletion of FTO leads to gene expression changes

**Figure 3.**
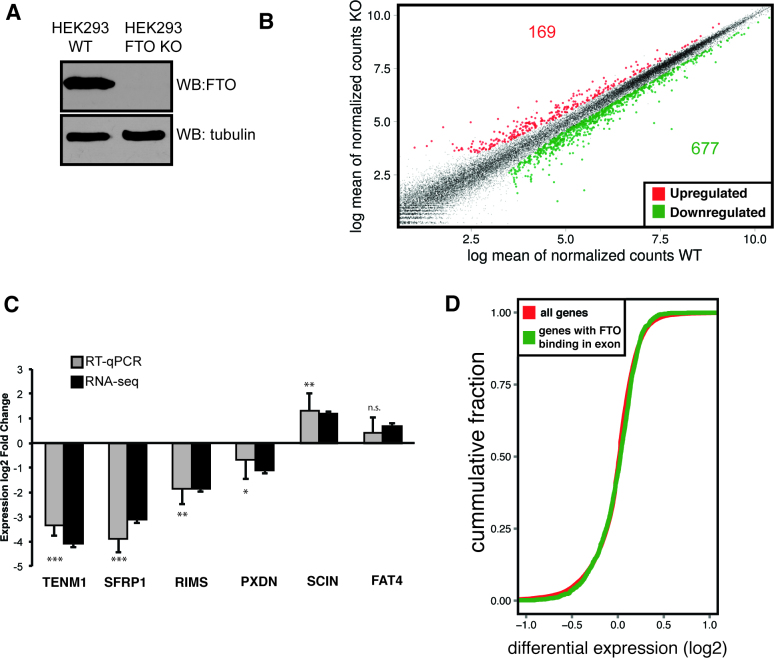
FTO KO cells display significant changes in expression of protein-coding genes (**A**) Western blot analysis of FTO expression in control 293T (WT) and FTO KO cell lines. (**B**) Scatter plot of gene expression in 293T (WT) and FTO KO cell lines. Significantly differentially expressed genes are colored with red (upregulated) and green (downregulated) (**C**) RT-qPCR analysis of gene expression for genes selected based on RNA-seq differential expression. Bars represent the mean of three biological replicates normalized to the expression of HPRT1 as an internal control and error bars represent standard deviation. Asterisks denote the significance of DE expression measured by qPCR: *P < 0.1, **P < 0.05, ***P < 0.001, n.s. not significant; *P*-values were calculated by a two-tailed paired Student's *t*-test (**D**) Cumulative distribution function of the differential expression of all genes (red) compared to FTO-bound genes identified by CLIP-seq (green).

Next, we asked whether mRNA binding by FTO or its demethylation activity could affect gene expression. We targeted exon 2 of *FTO* using a CRISPR-Cas9 system and generated a 293T *FTO*^−/-^ cell line (FTO KO) with abolished expression of both alleles of the *FTO* gene (Figure 3A). FTO KO cells displayed slower growth rate compared to the 293T control cell line (Supplementary Figure S3A). To investigate changes in gene expression, we performed 2 × 125 bp paired-end whole transcriptome sequencing (RNA-seq) of cDNA libraries prepared from rRNA-depleted total RNA with high sequencing depth. Gene expression was estimated using the R package DESeq2 (48). We observed differential expression (DE) of more than 0.5log_2_ fold in 846 protein coding genes (FDR < 0.05), of which 677 were downregulated in FTO KO cells, and 169 were upregulated (Figure 3B). We were able to validate the expression changes in either direction in five out of six selected mRNAs by RT-qPCR (Figure 3C). The DE data are consistent with the expected hypermethylation of mRNA in FTO KO and the known role of m^6^A in reduction of RNA stability (4).

We also investigated whether FTO-bound genes display a specific change of expression upon FTO deletion. To address this question, we compared the differential expression profile of genes bound by FTO with the differential expression profile of all genes (Figure 3D, Supplementary Figure S3B). Surprisingly, we did not detect a pronounced difference between the gene expression profiles. Thus, we concluded that regulation of gene expression and mRNA stability might not be the major role of nuclear FTO. We next focused on the possible role of FTO in mRNA processing.

### FTO triggers inclusion of alternatively spliced exons

**Figure 4.**
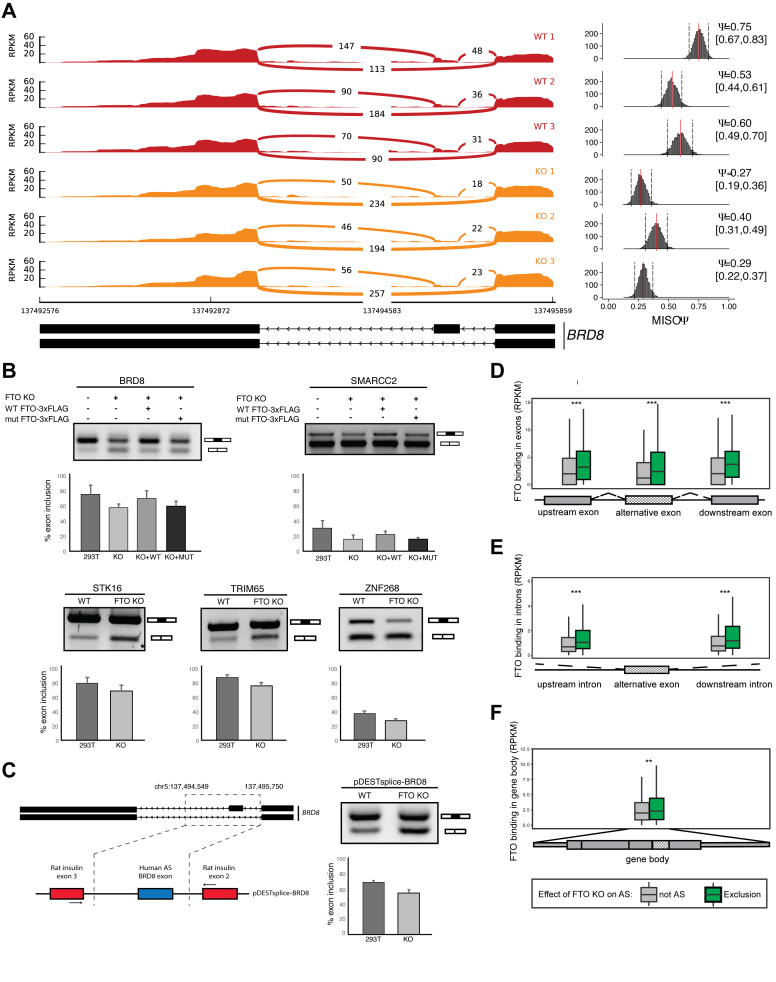
FTO depletion leads to exon skipping events. (**A**) Graphical representation of RNAseq (sashimi plot) in control and FTO KO cells in a region containing alternative isoforms observed in FTO KO. Histogram of PSI (Percent spliced in) calculated by MISO is shown in the right of the sashimi plot. Ψ represents mean value and 0.95 percent confidence interval is shown in the square brackets. A scheme showing the annotated alternative splice isoforms of *BRD8* mRNA is shown below the graphs. (**B**) Confirmation of the exon skipping events in FTO KO by RT-PCR. PCR primers anneal to exons flanking the skipped exon. The exon inclusion level was examined in 293T cells (WT), 293T cells with KO of both copies of FTO (FTO KO) and rescue cell lines with either WT FTO (KO+WT) or HADA mutant FTO (KO+MUT). (**C**) The alternative splicing phenotype in 293T cells upon FTO KO is reproduced with a minigene reporter construct (scheme is depicted on the left). The *BRD8* exon 20/21 inclusion level was analyzed by RT-PCR from 3 biological replicates. (**D–F**) Boxplots showing the metagene distribution of FTO binding (RPKM) in the proximity of the 200 most significantly skipped exons. Boxes represent binding in (D) flanking exons, (E) flanking introns or (F) the whole gene body. Asterisks denote significance **P < 1 × 10^−6^, ***P < 1 × 10^−8^. *P*-values were calculated by Mann–Whitney U-test.

FTO was previously linked to regulation of alternative splicing of a single gene RUNX1T1, which has an important role in adipogenesis (19,21). Furthermore, our CLIP-seq and RNA-seq data point towards a function of FTO in nuclear mRNA processing. To examine the role of FTO in alternative splicing globally we carried out exon expression analysis of FTO KO RNA-seq data with DEXSeq (49) and AS analysis with MISO (50). Both approaches revealed multiple differential splicing events relative to the control cell line (Table 1). Interestingly, these were predominantly exon-skipping events in FTO KO cells (957/1362 DEXSeq, 156/204 MISO; Table 1). We observed around 20% overlap of genes with significantly changed exon expression found by DEXSeq and significant difference of PSI estimated by MISO. A detailed view of a selected alternative splicing event (BRD8 gene) showing RNAseq read coverage across splice junction is represented in Figure 4A. Based on these analyses we selected six alternative splicing events and we were able to validate five of them using semi-quantitative PCR with primers annealing to flanking exons (Figure 4B). To further examine whether the demethylation activity of FTO, and thus the m^6^A, are involved in the regulation of the AS events, we stably reintroduced WT FTO and catalytically inactive mutant FTO (in α-ketoglutarate coordinating site H231A, D233A), respectively into the 293T FTO KO cell line. Whereas the WT FTO expression partially rescued the exon skipping phenotype of the FTO KO (Figure 4B), the splicing pattern in mutant FTO cells was comparable to FTO KO (Figure 4B).

**Table 1.** Summary of differential expression and splicing events found by MISO and DEXSeq. KO of FTO leads predominantly to skipping of internal cassette exons and upregulation of last exons

		Downregulated (skipping)	Upregulated (inclusion)
FTO	Internal exons	957	405
	MISO AS events	156	48
	Last exons	32	869
METTL3	Internal exons	1525	1061
	Last exons	498	48

To further validate the alternative splicing phenotype, we prepared a reporter minigene construct, in which the alternative exon of *BRD8* together with surrounding intronic regions was inserted between heterologous exons of the rat insulin gene (Figure 4C). The experiments with the reporter construct revealed 68% versus 54% exon inclusion in control 293T versus FTO KO cells, respectively, which is highly similar to the splicing patterns observed in endogenous *BRD8* (75% control and 57% in FTO KO, respectively).

To address whether FTO binding correlates with exon inclusion, we compared FTO CLIP-seq coverage around top 200 most significantly excluded alternative exons to the total set of 339881 cassette exons present in MISO splicing events database. Because MISO does not support replicates we pooled the three replicate libraries together and also performed the analyses separately for each replicate. Alternative splicing events were scored and selected from the pooled analysis. Individual AS events selected for validation were then examined in each replicate separately and reproducibility was tested by the t-test. We found the highest enrichment of FTO binding in both exons and introns adjacent to the excluded exons (Figure 4D, E). There was also a slight enrichment of FTO binding in the entire transcripts that contained excluded exons (Figure 4F). Conversely, we found that FTO binding was not significantly increased around exons that were more included in FTO KO (Supplementary Figure S4B). The CLIPseq results showed a strong enrichment of binding in the flanking introns of the BRD8 AS exon 20/21. Therefore, we used the reporter construct (Figure 4C) to try to narrow down the FTO target sequence. We selected a 53 nt long region in the downstream intron, that showed the highest FTO coverage and prepared mutant reporters lacking the whole region or replaced by a heterologous sequence (Supplementary Figure S4A). Interestingly, this 53 nt region contains two putative DRACH motifs. The mutant reporters showed higher exon inclusion than the wt form in FTO KO cells (Supplementary Figure S4A). A minor increase in exon inclusion from mutant constructs was also observed in control 293T cells, which is consistent with the model that absence of m6A marks inhibits exon inclusion in this AS event (Supplementary Figure S4A). In summary the minigene experiments supports our finding that the selected region is involved in AS of BRD8 exon 20/21.

To test whether the FTO-related alternative splicing events also correlate with m^6^A modification, we compared the m^6^A abundance around alternatively spliced exons. The m^6^A marks were strongly enriched at intronic as well as exonic regions surrounding skipped exons in FTO KO cell line, but not in the whole mRNA body (Supplementary Figure S4C). Interestingly, m^6^A was also not enriched around exons that were differentially included upon FTO depletion. Finally, transcripts with more included exons in FTO^−/−^ cells displayed overall higher level of m^6^A marks (Supplementary Figure S4C). In conclusion, the preferential binding around skipped exons and prevalence of exon skipping events in FTO KO, suggested that the demethylation of mRNA transcripts by FTO directly promotes exon inclusion under normal conditions.

Previous studies showed that transcripts of housekeeping genes are devoid of m^6^A marks (5). To address whether proteins encoded by the FTO-regulated alternative mRNA isoforms belong to specific cellular pathways, we performed a GO term enrichment analysis of the alternatively spliced transcripts. We used mRNAs that shared both features: revealed exon skipping in FTO KO cells and were identified by FTO CLIP-Seq analysis. The analysis showed a significant overrepresentation of genes encoding factors involved in cell cycle, nucleic acids binding and metabolism and general metabolic processes (Supplementary Figure S4D).

### FTO regulates expression of last exons

**Figure 5.**
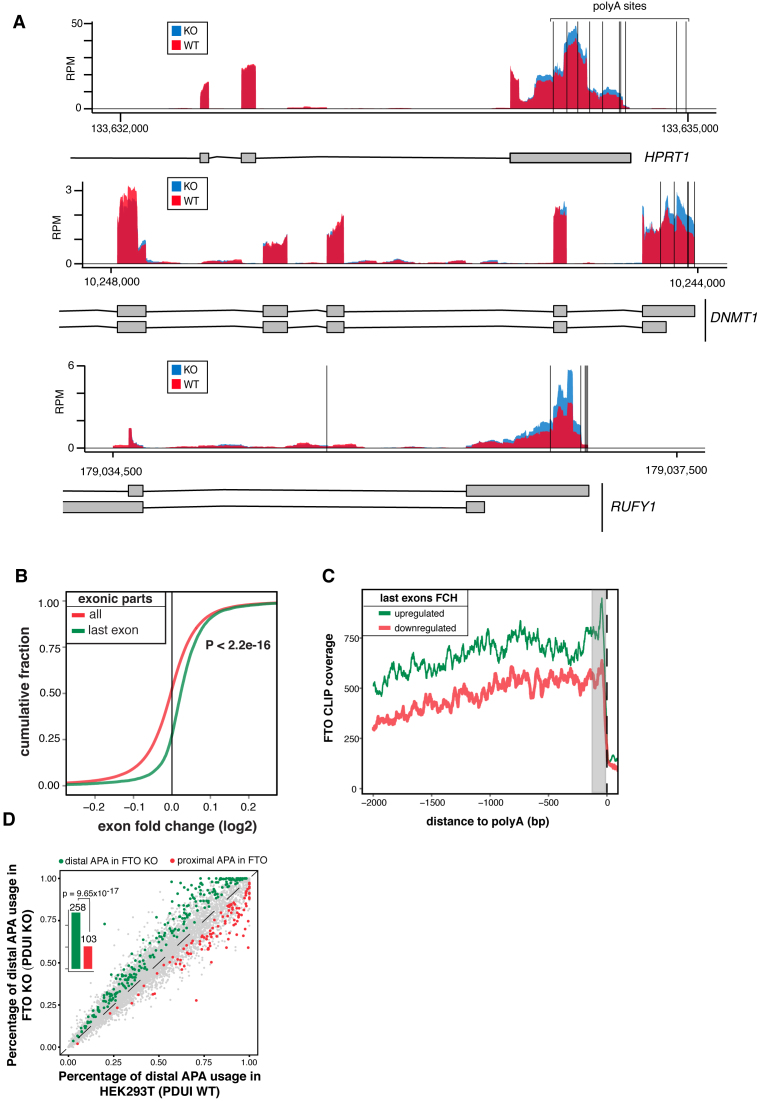
FTO KO leads to upregulation of extended 3′ UTRs (**A**) Genome browser examples of reads coverage in 293T WT and FTO KO cell lines in genes with changes in expression in the last exon. Vertical black lines represent the position of the annotated alternative poly(A) sites. (**B**) Cumulative distribution of expression of all exonic parts and last exons calculated by DEXseq. P value was estimated by Mann-Whitney U-test. (**C**) Metagene analysis of FTO CLIP read coverage around poly(A) sites of genes with upregulated (green) and downregulated (red) 3′ ends. (**D**) Scatterplot of percentage of distal poly(A) site usage index (PDUI) of 239T WT and FTO KO cell lines. Barplots summarize the number of APA usage events in FTO KO.

On top of the changes in AS, the DEXSeq analysis revealed that last exons underwent differential expression more frequently than expected by chance (Table 1; *P* = 1.62 × 10^−233^, Fisher's exact test). Differentially expressed last exons were almost exclusively upregulated (860 upregulated vs 32 downregulated; FDR < 0.05; Table 1, Figure 5A, B, *P* < 2.2 × 10^−16^ Mann-Whitney U-test), suggesting that FTO might play a role in regulation of expression and/or processing of mRNAs 3′ ends. To gain further insights into the profile of expression change at the 3′ mRNA termini, we performed a metagene analysis of 3818 transcript isoforms, which overlap with 892 DE 3′ terminal exons in FTO KO. We observed a strong increase in RNA-seq coverage in regions ranging from stop codons and covering the whole 3′ UTR in FTO KO compared to control cells (Supplementary Figure S5A).

Next we investigated the relation between FTO binding and DE of last exons. To examine the precise location of FTO binding in last exons, we performed metagene analysis of 1258 and 1298 RefSeq transcripts overlapping with downregulated and upregulated last exons, respectively. We found that FTO CLIP-seq signal shows a peak of enrichment close to the poly(A) sites for transcripts with upregulated last exons, compared to transcripts with downregulated last exons (Figure 5C). As expected, no difference in enrichment of FTO binding was observed around putative poly(A) sites of transcripts with DE internal exons (Supplementary Figure S5B). These findings suggested that m^6^A and FTO might be involved in the regulation of poly(A) site selection and potentially in the control of 3′ UTR length. This is in agreement with the recent study that revealed m^6^A enrichment in last exons and its proposed role in the regulation of 3′UTRs (32).

To examine the significance of the last exon expression in FTO KO cells, we investigated whether the change in RNAseq coverage in FTO KO might be associated with the usage of alternative polyA sites (APA) using daPars (55) (Figure 5A). Consistently with DEXseq analysis, FTO KO cells showed pronounced higher usage of distal APAs comparing to the control cells (Figure 5D). To further support the involvement of APA in FTO KO cells, we plotted the ratio of FTO KO/WT RNAseq coverage around the annotated proximal polyA sites (58). We found that the change in coverage was strongly associated with the most proximal polyA site in the last exon (Supplementary Figure S6A), but not with the most proximal polyA site upstream of the last exon (Supplementary Figure S6B). This indicated, that FTO promotes usage of proximal APAs in a subset of genes. Therefore we tried to look whether these positions are bona fide cleavage sites. For that, we used the PAR-CLIP results of several 3′ end processing factors, that were obtained from T293 cells (59) and plotted their binding around APA sites predicted by daPars (767 sites). We observed a discrete peak of binding of the Cleavage and Stimulatory Factor CSTF64 and Cleavage Factor I 68 at FTO-linked APAs (Supplementary Figure S7A). Altogether, these data support the direct role of FTO in selection of APA sites in human cell culture model.

### FTO and METTL3 act reciprocally in the regulation of alternative splicing and 3′ UTR expression

**Figure 6.**
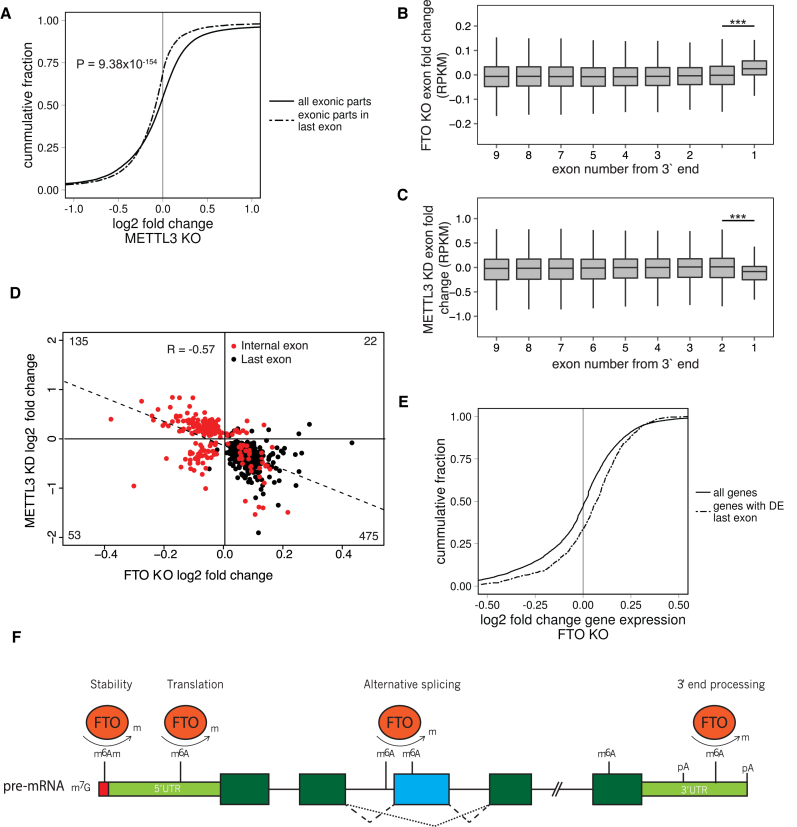
FTO and METTL3 deficient cell lines show anti-correlated 3′ UTR expression phenotype. (**A**) Cumulative distribution of expression of all exonic parts and exonic parts originating from last exons in METTL3 KD as calculated by DEXseq. *P* value is estimated by the Mann-Whitney U-test. (B, C) Boxplot showing differential expression of exonic parts in FTO KO (**B**) and METTL3 KD (**C**) based on the exon number from the 3′ end. Significance was calculated by a two-tailed t test. ***P < 1 × 10^−20^. (**D**) Scatterplot of expression change of exons that were called differentially expressed by DEXSeq in both FTO KO and METTL3 KD. R represents the Pearson correlation coefficient. (**E**) Cumulative distribution of differential expression of genes which have significantly changed expression of their 3′ terminal exonic part. P value is estimated by the Mann-Whitney U-test. (**F**). Schematic summary of the reported roles of FTO-dependent m^6^A and m^6^A_m_ modifications. Reversible m^6^A_m_ modification of the nucleotide adjacent to the 7-methylguanosine cap affects mRNA stability (60) and reversible m^6^A modification on the 5′UTR promotes cap-independent translation initiation under stress conditions (23–25). At the pre-mRNA body, FTO regulated m^6^A modification promotes exon inclusion/skipping, likely depending on the cellular context and interacting splicing factors (19,21). At the 3′UTR, FTO dependent m^6^A modification potentially regulates APA usage and the length of 3′UTR.

To investigate the relevance of m^6^A in FTO-dependent terminal exon upregulation, we examined the transcriptome analysis of cells with downregulated METTL3 (METTL3 KD) published by Liu *et al.* (7). METTL3 KD data displayed a significant downregulation of 3′ terminal exons (*P* = 9.38 × 10^−154^, Mann-Whitney U-test, Figure 6A, C), which is the opposite effect to the last exon upregulation in the FTO KO (Figure 6B). Overall, 88% of last exons (498/546) that were DE with at least 0.5log_2_ fold change were downregulated (Table 1). On the other hand, the effect on internal exons was not strictly unidirectional in METTL3 KD (Figure 6C). Most importantly, we observed an inverse correlation between the exon expression in FTO KO and METTL3 KD cells for both internal and terminal exons, supporting the functional involvement of the m^6^A pathway (Figure 6D, *R* = 0.57, Pearson correlation). Finally, we aimed to examine the global expression of genes with DE terminal exons in the FTO KO cell line. We selected 938 genes showing significant (FDR < 0.05) DE of their 3′ exons and plotted their change of gene expression in comparison to the change of expression of all genes in the FTO KO cell line. We found a significant upregulation of expression of genes with upregulated 3′ ends (Mann-Whitney U-test *P* = 1.13 × 10^−18^, Figure 6E). In conclusion, these data revealed an opposite effect of METTL3 and FTO in regulation of expression of internal and 3′ terminal exons, which strongly supports the role of m^6^A methylation pathway in the regulation of 3′ mRNA processing and alternative splicing.

## DISCUSSION

The enrichment of m^6^A marks can have significant consequences on multiple aspects of mRNA metabolism. Despite the increasing number of m^6^A studies, it is still unclear why adenosines are preferentially modified at specific locations within transcripts, or what are the functional consequences of such patterns of methylation. It is tempting to speculate that demethylases are important players that decide about the final m^6^A pattern. Here, we report a transcriptome-wide mapping of the FTO m^6^A demethylase binding sites on RNAs and reveal the connection between FTO binding, activity and pre-mRNA processing.

In our model system, FTO is primarily localized to the nucleoplasm, where it appears to modulate several pre-mRNA processing events. FTO binding sites partially overlap with m^6^A-associated features, such as TSS and stop codons (1,2), which experimentally demonstrates the predicted effect of demethylation on m^6^A abundance at these sites. In mRNAs, m^6^A is frequently enriched in the DRACH consensus motif. Surprisingly, our CLIP-seq experiment revealed no significant enrichment of DRACH among FTO targets. We hypothesize that FTO may remove adenosine methylation marks also on non-DRACH sequences, and thus contributes to the pattern of m^6^A distribution. A recent study indicated that FTO and ALKBH5 discriminate their m^6^A targets based on structural rather than primary sequence properties *in vitro* (38). However, we have not observed higher folding energies in FTO-bound regions. Moreover, in the course of the revision of this manuscript, the work of Mauer *et al.* (60) uncovered, that FTO preferentially demethylates non-DRACH N6,2′-O-dimethyladenosine (m^6^A_m_) present downstream of the mRNA 5′ cap. This well correlates with the FTO binding enrichment around TSS observed in our datasets (Figure 1C).

Adenosine methylation is a dynamic process that among others contributes to the flexible regulation of gene expression at the level of mRNA stability (3,4,8). The m^6^A marks in the 3′ UTRs promote mRNA destabilization via the recruitment of the cytoplasmic YTHDF2 m^6^A reader (4). However, our data suggest that the main role of nuclear FTO is regulation of pre-mRNA processing rather than regulation of mRNA expression. This hypothesis is supported by the large proportion of intronic and exon-intron boundary reads in the FTO CLIP-seq as well as the absence of correlation of FTO binding and gene expression. Additionally, studies investigating the mRNA interactome identified components of the methylase complex (METTL3, KIAA1429) and all known YTH proteins (YTHDF1, YTHDF2, YTHDF3, YTHDC1, YTHDC2) as poly(A)-RNA interacting proteins (61,62). FTO was not detected in the poly(A) RNA interactome suggesting that it does not interact with mature mRNA. Taken together, we propose pre-mRNA as the major substrate of FTO.

Recent work of Ke *et al.* suggested that higher m^6^A density in last exons could influence the usage of alternative poly(A) sites (APA) (32). Notably, our CLIP-seq analysis revealed a substantial enrichment of FTO binding in upregulated last exons, which was particularly pronounced in the close proximity to poly(A) sites. Moreover, we demonstrated, that FTO acts at APAs that are actively used and regulated in the 293T cells. In this context, the upregulation of last exons in FTO KO cells points to the role of methylation dynamics in regulation of APA usage and in turn control of 3′ UTR length.

Several lines of evidence established the m^6^A as an important pre-mRNA splicing regulator. WTAP was originally known as a splicing factor in *Drosophila* (63) and downregulation of several players in the m^6^A pathway was previously shown to affect the AS pattern of thousands of genes (18,20,21). The data presented in this work further demonstrated that FTO binds pre-mRNAs *in vivo*, allowing for AS regulation and that FTO depletion causes mainly exon skipping phenotypes. These conclusions are supported by data from FTO CLIP-seq, RNA-seq of WT and FTO KO cell lines and by subsequent RT-PCR validation on several endogenous transcripts, as well as a reporter construct. To narrow down the FTO-regulated RNA element, we introduced mutations in the FTO binding region, which partially reverted the alternative splicing phenotype of FTO KO. However, since FTO CLIP-seq data showed additional peaks across the AS exon and flanking introns, we conclude that the effects of elements responsible for the AS regulation are additive and cannot be fully abolished by mutating only a single site.

FTO binding primarily to pre-mRNA intronic regions resembles the CLIP-seq results of METTL3-METTL14 published by other groups in which they reveal mainly intronic occupancy (29–34%) (7). Given its nucleoplasmic localization, FTO appears to also act on pre-mRNAs in concert with the methylase complex. These data support the hypothesis that a large number of intronic adenosines are methylated co-transcriptionally, enabling potential regulation of nuclear events. This would allow the regulation of alternative splicing events observed in our RNA-seq analysis of FTO KO cells. Because FTO depletion leads mostly to exon skipping AS events and FTO binding is enriched around skipped exons, demethylation appears to be important to facilitate exon inclusion in a subset of AS regions. Interestingly, several recent studies proposed novel m^6^A-dependent molecular mechanisms regulating alternative splicing. The direct recognition of m^6^A-enriched AS exons by the reader YTHDC1 facilitates their inclusion into mRNA. YTHDC1 recruits the SRSF3 splicing factor, which promotes exon inclusion and simultaneously prevents binding of SRSF10, which drives exon exclusion. As a consequence, 160 long AS exons are included into mature mRNA (18). A similar subset of AS exons is then excluded in the absence of m^6^A. Another m^6^A mechanism to regulate AS is based on RNA conformational changes induced by the modification. The m^6^A-mediated structural switch enhances binding of the splicing factor hnRNPC (20), which in turn affects splicing of adjacent exons (20). A parallel study showed that accumulation of m^6^A marks upon FTO depletion in mouse pre-adipocytes promotes binding of another splicing factor, SRSF2, which leads to increased inclusion of target exons (21). The fact that we observe an opposite trend upon FTO depletion in 293T cells suggests that FTO-mediated m^6^A enrichment leads to altered binding of different splicing factors depending on the cellular and mRNA context. This would resemble the opposing roles on SRSF3 and SRSF10 factors. Notably, studies in the *Drosophila* system by (64–66) show that m^6^A deficient ΔIme4/Yt521-B mutant flies exhibit aberrant exon inclusion in Sxl exon3. This is consistent with exon skipping phenotype observed in FTO KO human cell lines and association of FTO activity (which reduces m6A content) with exon inclusion.

Recent work from Ke *et* al. (67) uncovered that m6A deposition occurs mostly on chromatin-associated pre-mRNAs prior to the completion of pre-mRNA processing and the overall m6A deposition does not change after the mRNA is released from chromatin. They observed the highest density of m6A in exonic regions even though introns represent majority of pre-mRNAs and contain higher proportion of DRACH motifs. This is consistent with our findings, that FTO binds to pre-mRNAs and mediates m^6^A removal in introns. The major conclusion of Ke *et* al. study points to the role of m^6^A mostly in regulation of mRNA stability (67). Nevertheless, the transcriptome-wide analyses of splicing patterns in wild type and METTL3 KO ESCs and also reanalyzed datasets from (2) (20,21) showed that perturbation of m^6^A causes a minor, but genuine effect on splicing of alternative exons with m^6^A modifications (67). This is in agreement with our conclusions on the role of FTO in regulating alternative splicing of a subset of exons in HEK293T cells.

The inconsistency between published reports illustrates, that the mechanism of m^6^A in regulation of constitutive and alternative splicing is not well established, yet. Certain discrepancy in this context might be caused by selective analyses of isolated effects of m^6^A on splicing in the context of other splicing factors. Although, in specific cases, m^6^A promotes exon inclusion (18) or exon exclusion (64–66), drawing conclusions about general effect of m^6^A on AS might not be relevant. It rather seems that the effect of m^6^A addition/removal is transcript-specific and depends on particular downstream effectors.

Finally, the removal of m^6^A by FTO is a multi-step process. It can generate up to two intermediates (*N*^6^-hydroxymethyladenosine (hm^6^A) and *N*^6^-formyladenosine (f^6^A)) with expected lifetimes of around 3 hours (68). It means that these intermediates could also be recognized by different factors and change the fate of the modified transcripts. Moreover, in each of the above-mentioned studies, only a small subset of exons reacted to the particular splicing factors. It is likely that m^6^A affects multiple independently acting factors and thus can have adverse consequences. This is supported by finding of Xiao *et al.* who showed that YTHDC1 does not directly interact with another m^6^A-responsive splicing factor, such as hnRNPC or ELAVL1 (18). The identification of m^6^A_m_ as a new target of FTO adds to the complexity of the picture (60). Unfortunately, we do not know which additional splicing factor responds to m^6^A or m^6^A_m_ enrichment upon FTO depletion. However, our data for the first time demonstrate the direct link between FTO activity, mRNA binding and mRNA processing consequences.

The m^6^A and m^6^A_m_ decoration of RNA is a dynamic process with a wide spectrum of consequences, which have been connected to many important mRNA metabolic processes. However, the mRNA demethylation still remains one of the most elusive steps in the RNA methylation pathway. In this study, we demonstrated that RNA demethylase FTO acts primarily in the nucleus, where its demethylation activity regulates pre-mRNA processing including alternative splicing and 3′ UTR processing (Figure 6F). These results add an additional component to the mosaic of adenosine methylation-regulated processes in mammalian cells.

## DATA AVAILABILITY

Raw and processed CLIP-seq and RNA-seq data can be found in GEO database under accession code GSE79577.

## Supplementary Material

Supplementary Data
