# Supplementary material for: N6-methyladenosine demethylase FTO targets pre-mRNAs and regulates alternative splicing and 3′-end processing
Source: Nucleic Acids Res. 2017 Aug 30;45(19):11356–70. doi: 10.1093/nar/gkx778 (PMC5737695; doi:10.1093/nar/gkx778)
Supplement: Supplementary Data [file gkx778_supp.pdf]

Supplementary Figure 1

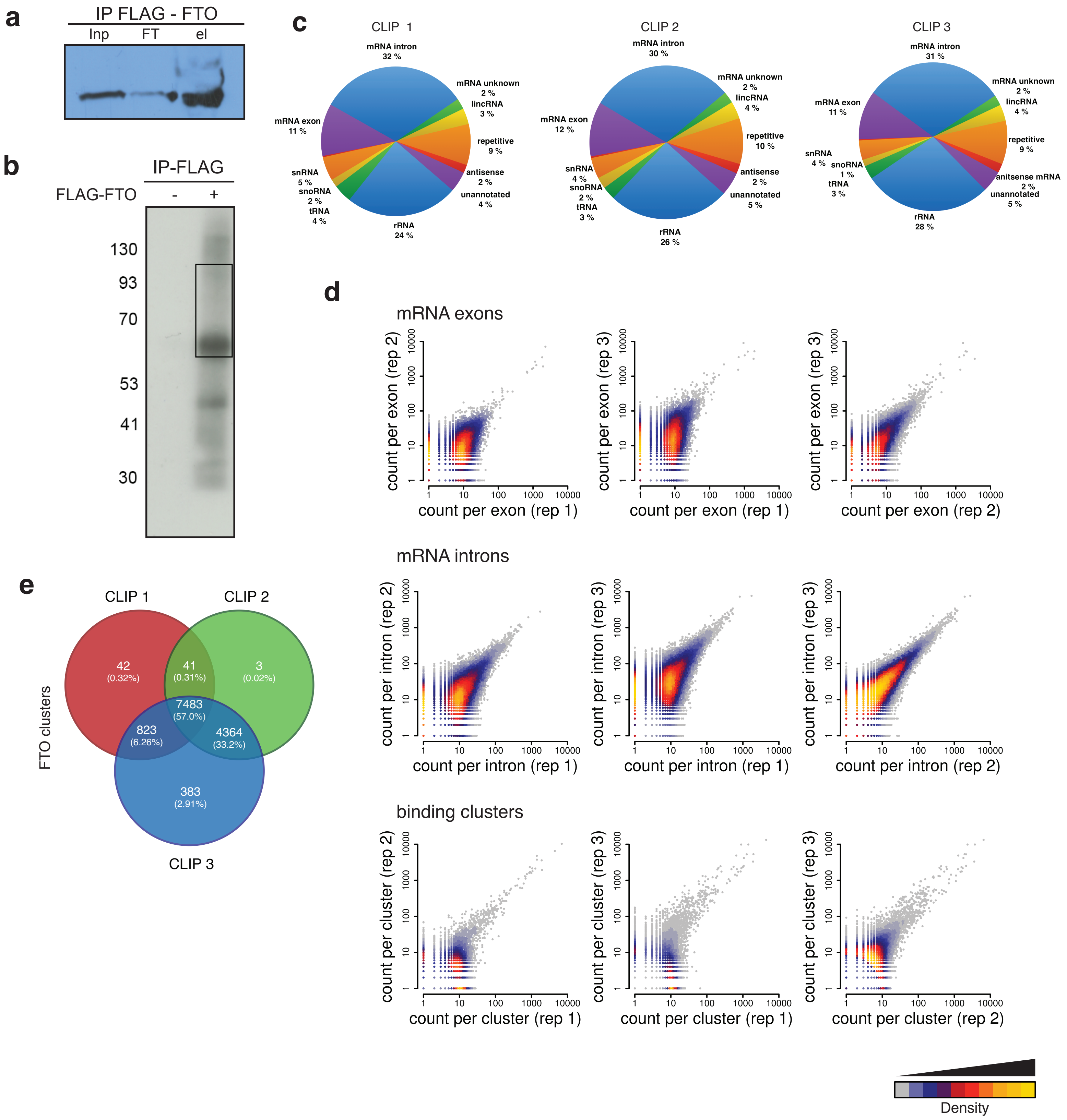

Supplementary figure 2

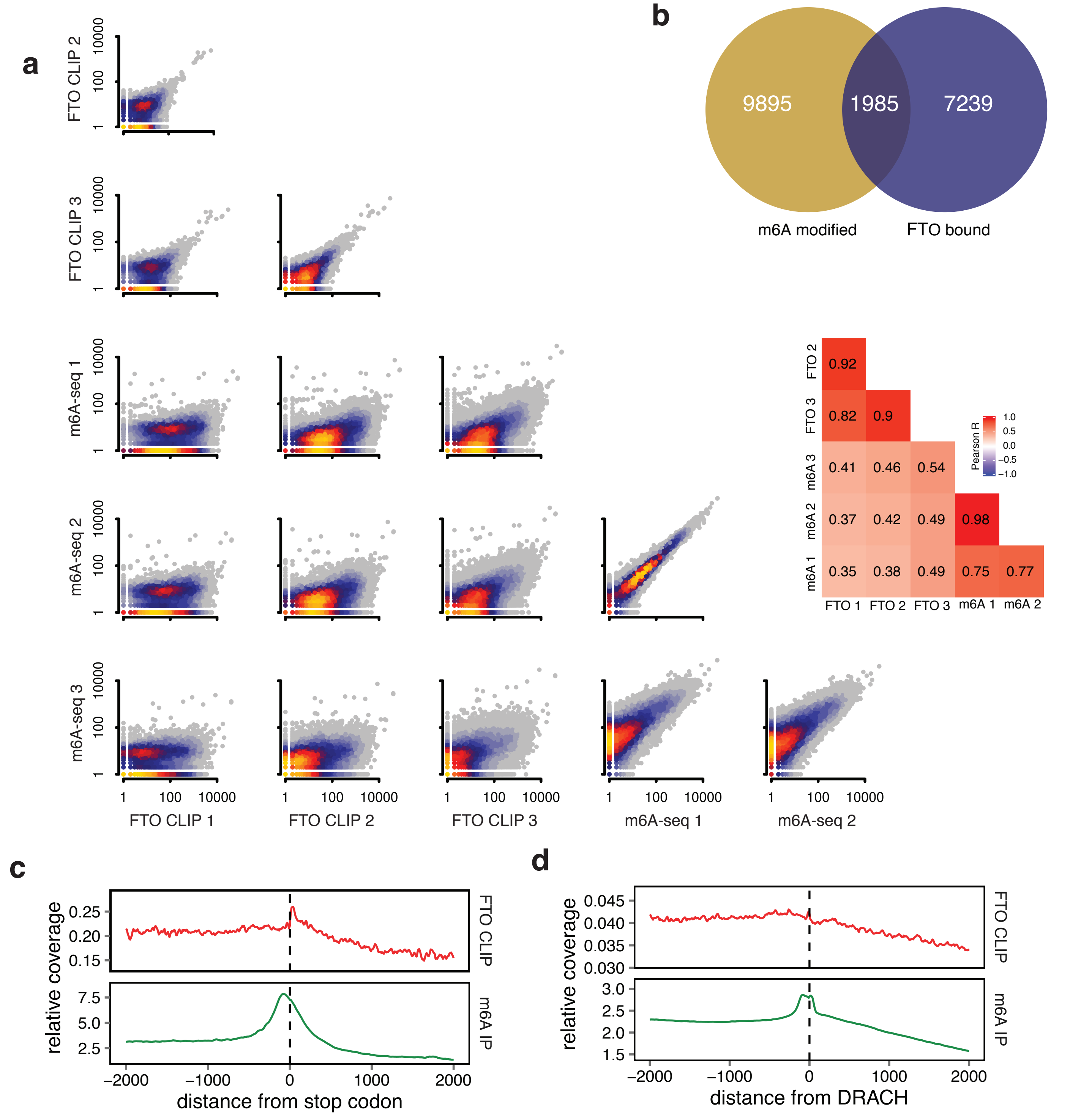

e

k-mer	enrichment (log2)	Adjusted p value	Z-score	Percent of sequences
CTTTT	1.90	1.12×10^{-81}	21.2	5.25 %
GTGTG	1.79	7.46×10^{-68}	19.6	5.19 %
CCTCT	1.67	4.29×10^{-59}	18.4	4.28 %
CTCTC	1.69	2.05×10^{-54}	17.6	3.92 %
TTTTG	1.53	1.43×10^{-50}	17.4	4.45 %
GCTTT	1.95	9.07×10^{-51}	16.9	3.44 %

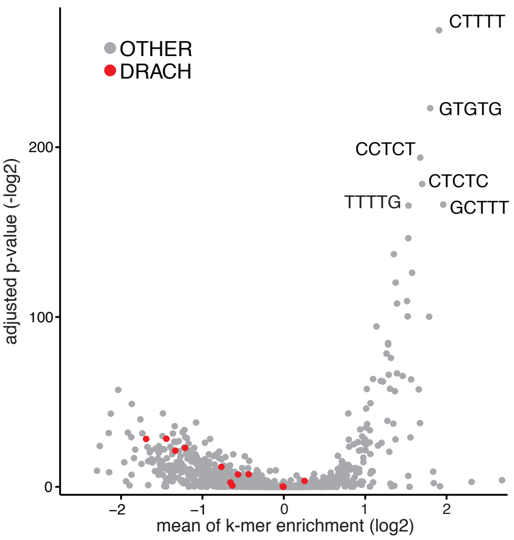

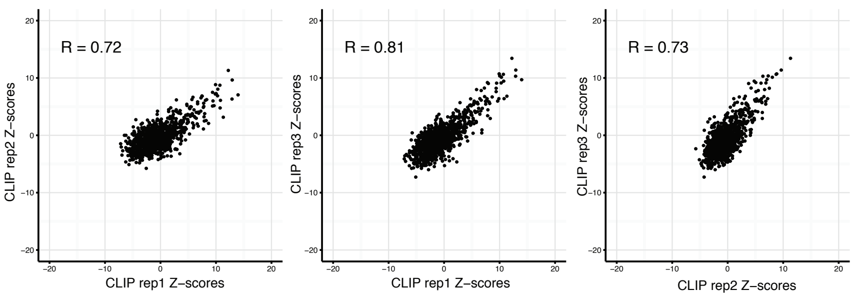

Supplementary figure 3

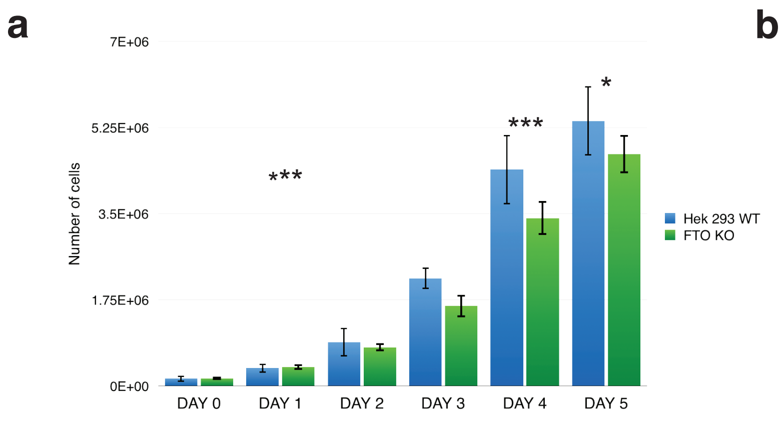

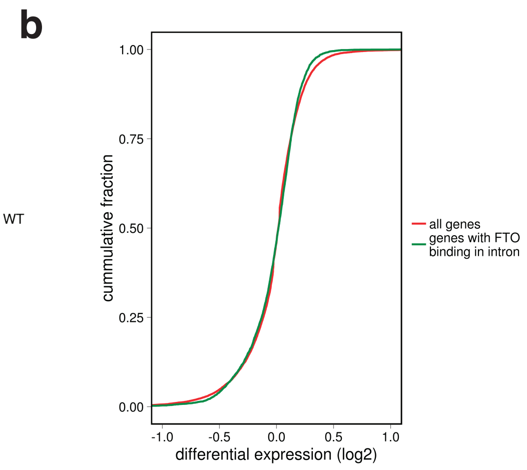

Supplementary figure 4

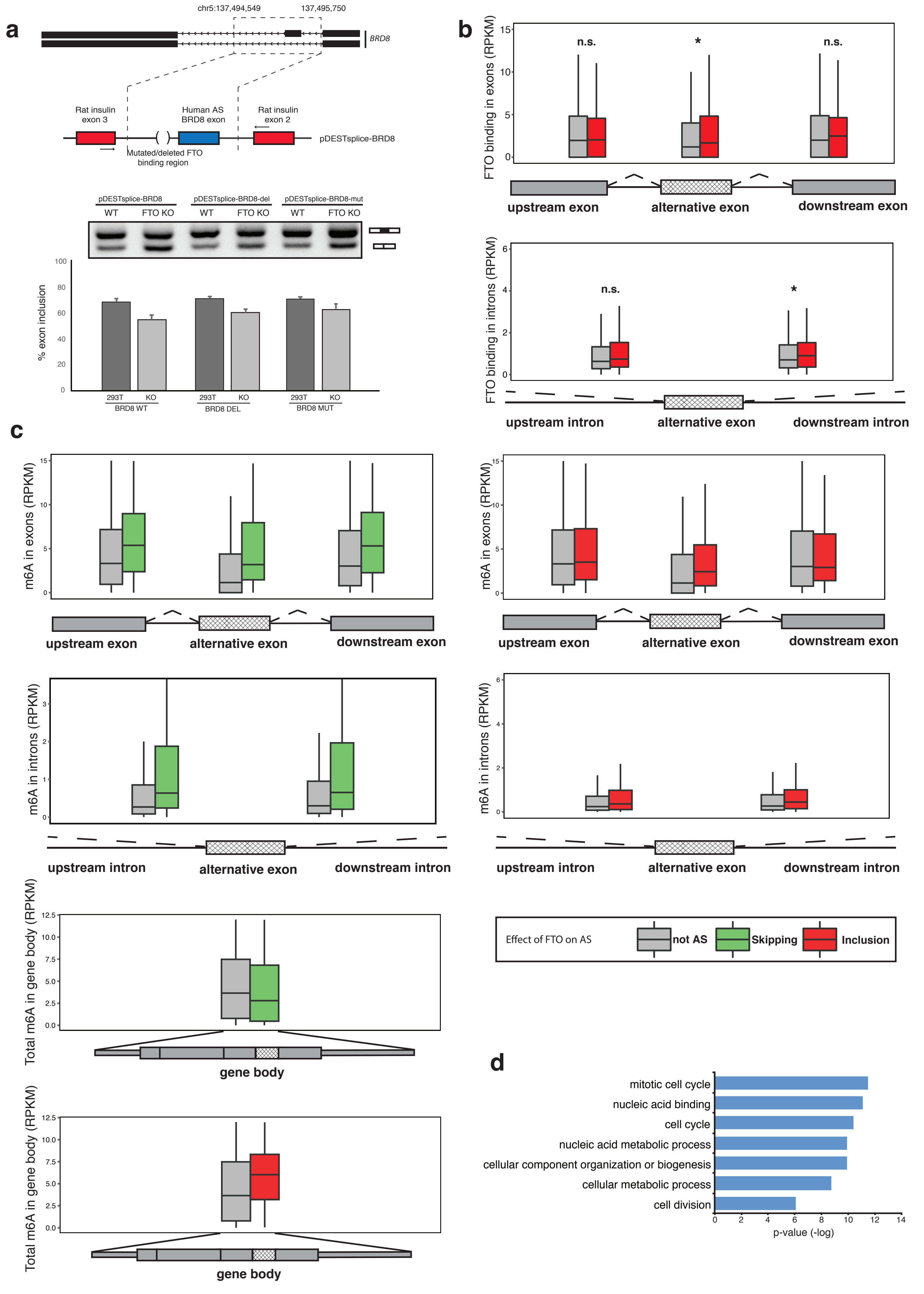

Supplementary Figure 5

a

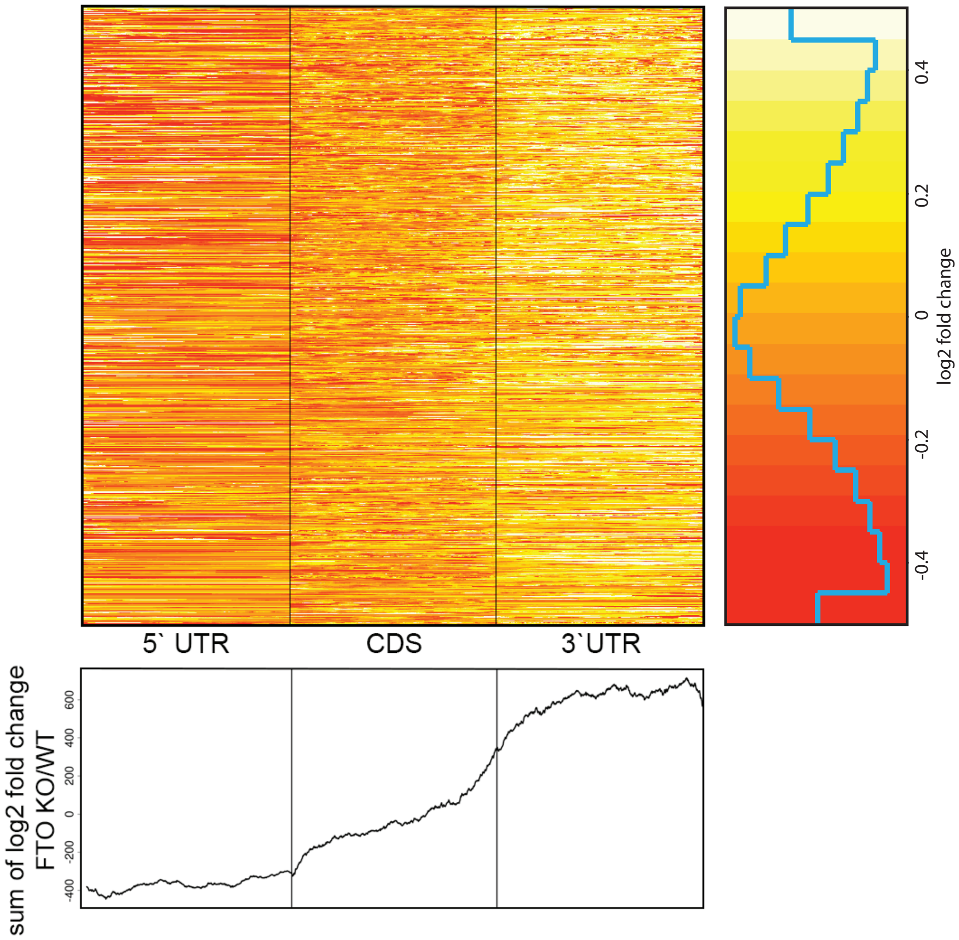

b

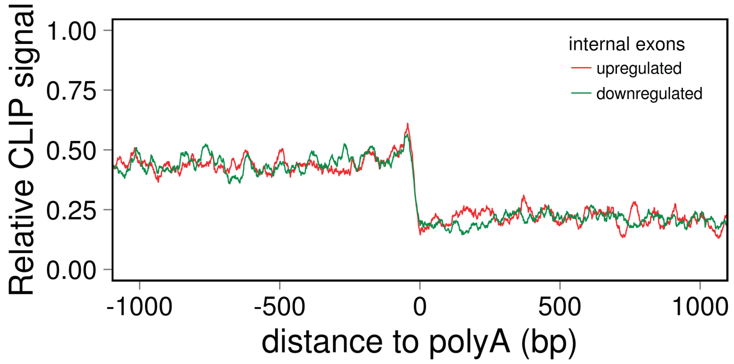

Supplementary figure 6

a

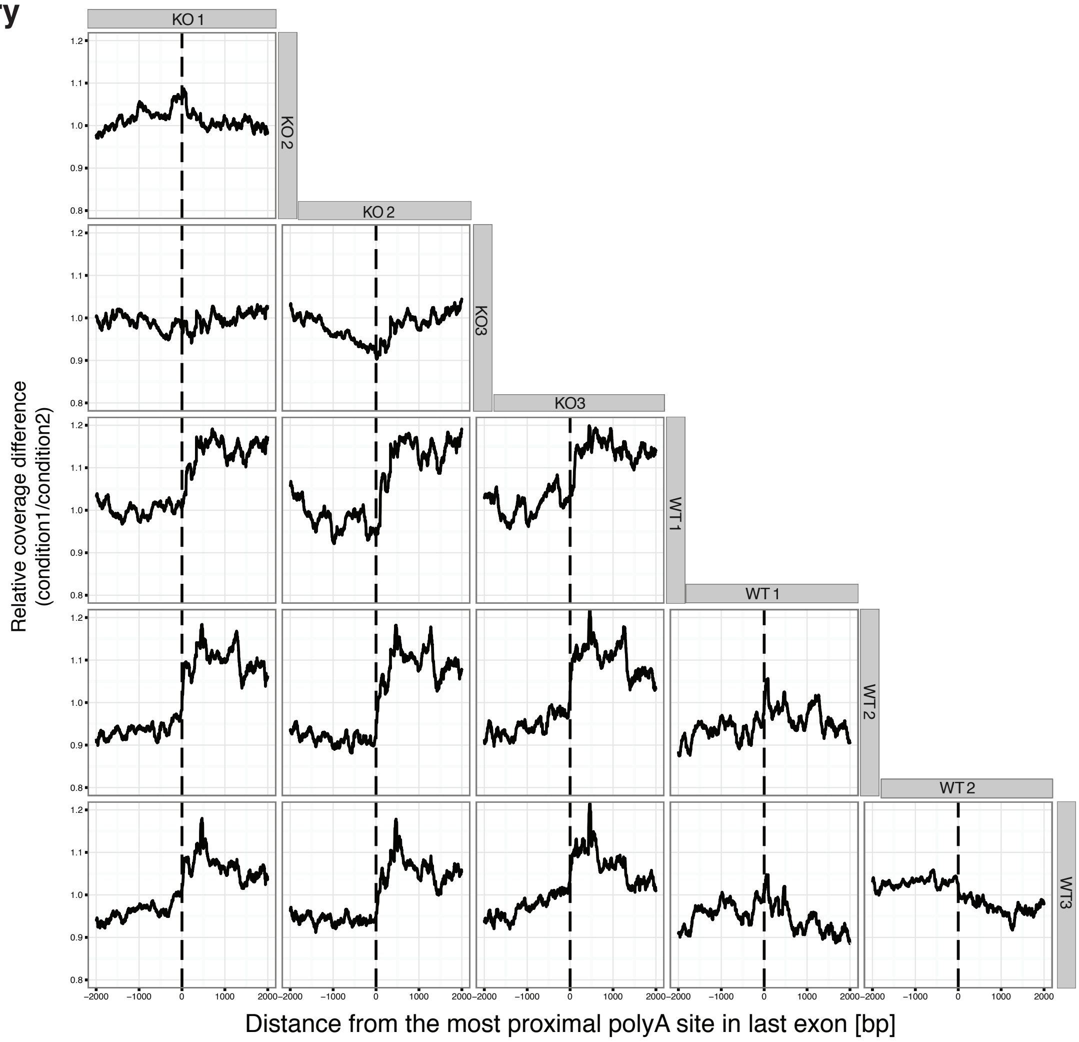

b

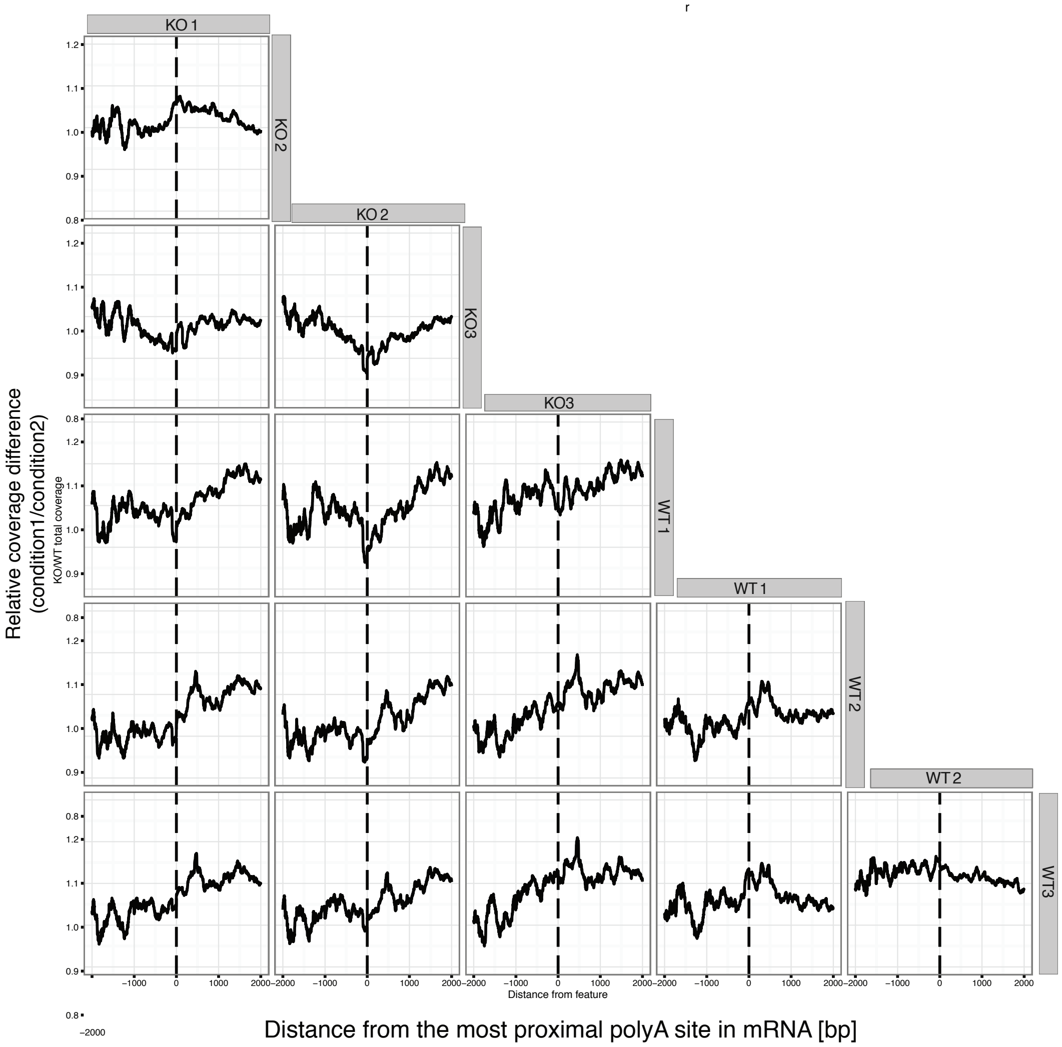

Supplementary figure 7

a

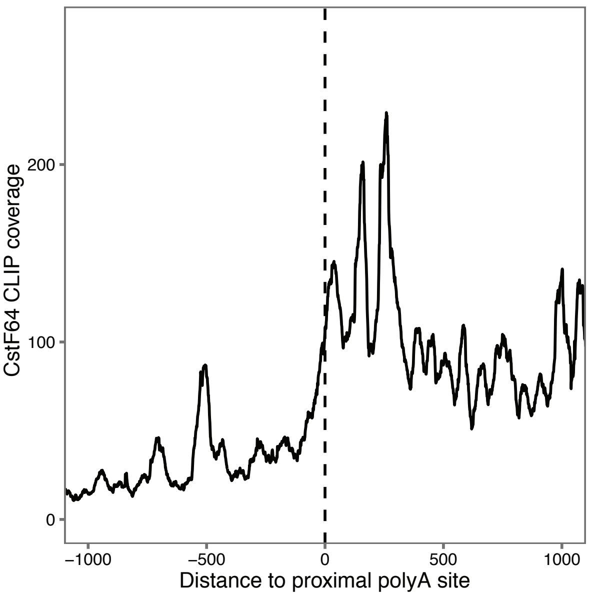

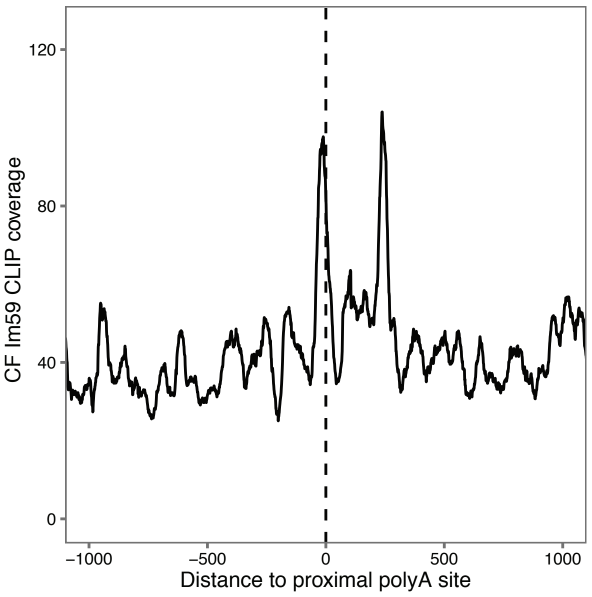

Supplementary table 1

FTO_fw_notI	GATGCGGCCGCAAGCGCACCCCGACTG
FTO_rev_XhoI	CGACTCGAGCTAGGGTTTTGCTTCCAG
FTO_HDmut_fw	GGGGAAAATGGCAGTGAGCTGGGCTCATGCTGAAAATCTGGTGGACAGGTCAGC
FTO_HDmut_rev	GTTTTGGCCGGTTCACAACCTCAGTTTAGTTCCACCCACCGAGTGGC
FTO_crispr_ex2-1_fw	CCACCGTTGAAGAGCTTGAAGACACT
FTO_crispr_ex2-1_rev	AAACAGTGTCTTCAAGCTCTTCAAC
FTO_crispr_ex2-6_fw	CACCGCATCTTTGGGGGTCAGATAA
FTO_crispr_ex2-6_rev	AAACTTATCTGACCCCCAAAGATGC
CLIP L3 adaptor	/5rApp/AG ATC GGA AGA GCA CAC GTC T/3ddC/.
CLIP L5 RNA linker	GUUCAGAGUUCUACAGUCCGACGAUC
RT_CLIP2	AGACGTGTGCTCTTCCGATCT
PCR3_CLIP_bcd1	AGAAGACGCATACGAGATCGTGATGTGACTGGAGTTCAGACGTGTGCTCTTCCGATCT
PCR3_CLIP_bcd2	[illegible]AGAAGACGCATACGAGATACATCGGTGACTGGAGTTCAGACGTGTGCTCTTCCGATCT
PCR3_CLIP_bcd3	AGAAGACGCATACGAGATGCCTAAGTGACTGGAGTTCAGACGTGTGCTCTTCCGATCT
PCR5_CLIP	AATGATACGGCGACCACCGACAGGTTCAGAGTCTACAGTCCGA
SRSF2_fwd	GGAACAAGCAACTGGCTATTGA
SRSF2_rev	GAAATCTGAAGTCGTTCACCTC
CARD6_fwd	AGATCTCCTGAAGAAAAGTCGG
CARD6_rev	AACTTCATGCCTTAAGCCGC
PIGM_fwd	ACATCTACCTCAGCGAGCTC
PIGM_rev	CCAAAAGACACAGTAGCCAC
HIST2H4B_fwd	TCCAGCTTTGCACGTTTCGAT
HIST2H4B_rev	AACTTCAGATCCGATTGTCGCC
sca12_f1	CAGGCTGATGAGACTAAGGC
sca12_r1	ATTGATCTGGCCTTACAGTG
sca10_f2	CTGGAGTGAGAGAGTAGGAA
sca10_r2	AGCCATCAGATTACCAAAGA
TENM1_qPCR_fwd	CTATAATCTGGCCTTAATGACC
TENM1_qPCR_rev	TGGGAAACGTTGCATTGGTCAG
SCIN_qPCR_fwd	AATGCTGCTGTCTGAAGAATG
SCIN_qPCR_rev	CTTCAGCTGTCTTCATTGCAG
RIMS2_qPCR_fwd	TCAGGTAGGAATGATGGACAAA
RIMS2_qPCR_rev	TGGCTATGCAGACTCCGTTATC
FAT4_qPCR_fwd	TTTGACATTCCTGAGGACAC
FAT4_qPCR_rev	ATGCCATCTTCATCATCTTC
SFRP1_qPCR_fwd	ACGAGTTGAAATCTGAGGC
SFRP1_qPCR_rev	GCTTCTTCAGGTCCTTCTTC
PXDN_qPCR_fwd	AGCGACACAACAGTGGAGG
PXDN_qPCR_rev	AATCCTTCAGGGCTGATGTG
BRD8_fw	GAGAGATTCTACCCGCAAACAGG
BRD8_rev	TATCTGCTTCAATGGCACAGCGG
STK16_fw	CTGGCTGCTGCTACCATTCTTC
STK16_rev	TCCATTAAAACTGGCTGCCCCTC
SMARCC2_fw	ACTTCCATGTCTTGGCTGACAC
SMARCC2_rev	GGTTTCTCTTTGCCTTTGTCAGG
ZNF268_fw	CAAGTTCCAAATCAGACCTGTCC
ZNF268_rev	CCTGATGCCAATCCATAAGATC
pSplice-Rat-Insuline exon 2_fw	CTGCTCATCCTCTGGGAGCC

Supplementary Figures legends

Supplementary Figure 1. FTO CLIP-seq analysis. (A) Western blot analysis of the efficiency of immunoprecipitation in the CLIP experiment. (B) Autoradiography of RNA-protein complexes purified during the CLIP-seq. Area above molecular weight of the protein, which is depicted by the rectangle, was used for sequencing library preparation. (C) Piecharts of the distribution of reads in RNA classes in the individual CLIP replicates. (D) Scatterplots of FTO CLIP showing the correlation of read counts in exons, introns and FTO binding clusters. (E) Venn diagram depicting an overlap of unfiltered FTO binding clusters. Clusters with at least 10 reads were selected.

Supplementary Figure 2. Comparison of FTO binding with m^{6}A-seq data. (A) Scatterplot of number of reads in exons for FTO CLIP and m^{6}A seq experiments. On the right is a heatmap with Pearson's correlation coefficient. (B) Venn diagram of the overlap of FTO bound exons and m^{6}A modified exons. (C, D) Metagene plots showing the distribution of FTO and m^{6}A-Seq reads around the stop codon (C) and DRACH sequences in exons (D), respectively. (E) Sequences identified by the motif search analysis of FTO binding sites defined with single-nucleotide resolution by deletions in the CLIP reads. Scatterplots show the correlation of the k-mer z-scores in the three CLIP replicates. R represents the Pearson's correlation coefficient. The volcano plot on the right illustrates the pentamer enrichments and p-value determined by the Fisher's exact test.

Supplementary Figure 3. Characterization of FTO KO cell line and gene expression analysis (A) Growth analysis of FTO KO and control 293T cell lines. Bars represent mean of 3 biological replicates and error bars are standard deviations. Significance was tested by the two tailed Student's t-test. *** $P < 0.001$; * $P < 0.1$ (B) Cumulative distribution of gene expression change (log2) of all genes and genes with FTO binding in introns.

Supplementary Figure 4. Correlation of alternative splicing in FTO KO, FTO binding and RNA methylation. (A) Schematic representation of the BRD8 gene region including AS exon 21 and surrounding intronic sequences, which was inserted between exons 2 and 3 of the rat insulin gene and transiently expressed from pDEST vector in 293T cells. The percentage of BRD8 21 exon inclusion was determined by RT-PCR in WT and FTO KO cells. (B) Boxplots of FTO binding around exons, which are more included in FTO KO. Asterisk marks the significance * $P<0.05$, n.s. – not significant. P-values were calculated by Mann-Whitney U-test. (C) Boxplot of RNA methylation levels around exons which are more skipped or included in FTO KO. (D) GO enrichment analysis of genes, which show exon skipping in FTO KO and are bound by FTO.

Supplementary Figure 5. FTO KO causes upregulation in the 3' UTR. (A) Heatmap illustrating the log2 fold change of expression in transcripts scaled to the same length and dissected to 5`UTR, CDS and 3`UTR. Each line represents one mRNA transcript. Only transcripts with differentially expressed last exons are displayed (n=3818). Sum of log fold change is shown below the heatmap. (B) Metagene plot of FTO binding around poly(A) sites in transcripts with DE (upregulated and downregulated) internal exons.

Supplementary Figure 6. Upregulation of mRNA 3' ends is associated with first proximal poly(A) site in the last exon. (A) Metagene plots showing the ratio of individual 293T WT and 293T FTO KO RNAseq coverages in transcripts with differentially expressed last exons (n=3818) around (A) most proximal poly(A) site in last exon (B) first poly(A) site in mRNA upstream of the last exon.

Supplementary Figure 7. mRNA cleavage factors bind in the proximity of poly(A) sites predicted by daPars. (A) Metagene plot of CstF64 and CF Im59 around proximal poly(A) sites predicted by daPars, based on change of RNAseq coverage in FTO KO relative to 293T WT cells.

Supplementary Table 1. The list of DNA and RNA oligonucleotides used in this study. The sequences of oligonucleotides are in the 5'to 3' orientation. Fw is forward, rev is reverse primer, bcd stands for an oligo introducing a sequencing a barcode.

Supplemental methods

Complete CLIP protocol

HEK293 Flp-In T-REx cells were grown to 40% confluency and induced with 0.1 mg/ml doxycycline (Applichem) overnight. Next day, cells were washed, UV-irradiated with 2x200 mJ/cm^2 and lysed in lysis buffer containing 100 mM NaCl, 50 mM Tris-HCl pH 7.4, 1% NP-40, 0.1% SDS, 0.5% Sodium deoxycholate and EDTA-free Complete Protease Inhibitor Cocktail (Roche). Lysates were sonicated briefly, treated with 10 ml of Turbo DNAse (Fermentas) and 10 ml of 1:1000 diluted RNAse I (Ambion) shaking at 37°C for 3 minutes and cleared by centrifugation. Cleared lysates were mixed with a-FLAG antibodies (Sigma) coupled to protein-G dynabeads (ThermoFisher Scientific) according to manufacturer's instructions and incubated for 1 hour rotating at 4°C. Beads were washed 3 times with CLIP high salt buffer containing 1M NaCl, 50mM Tris-HCl pH 7.4, 1mM EDTA, 1% NP-40, 0.1% SDS and 0.5% Sodium deoxycholate and 2 times with PNK wash 20mM Tris-HCl pH7.4, 10mM MgCl2, 0.2% Tween 20. Next, RNA was dephosphorylated on beads using Fast AP alkaline phosphatase (Fermentas) for 20 minutes at 37°C and L3 DNA adaptor was ligated using truncated T4 RNA ligase 2 (NEB) overnight at 16°C. Beads were then washed again 3x with high salt wash and 2x with PNK. RNA was then labeled using T4 PNK (NEB) and g-^{32}P ATP for 20 minutes at 37°C. Further 2 ml of cold 10mM ATP (NEB) was added afterwards and reaction was incubated for 15 minutes at 37°C. Beads were washed again 2x with PNK buffer and eluted by boiling for 5 minutes in 1x NuPAGE LDS buffer (ThermoFisher Scientific). Elution was separated on NuPage 4-12% gel (ThermoFisher Scientific) and blotted onto nitrocellulose membrane (BioRad). Protein-RNA complexes were visualized by autoradiography and corresponding range of molecular weights was excised from membrane. RNA was extracted by proteinase K, phenol/chloroform extraction and precipitated with ethanol. The second RNA linker L5 was ligated using T4 RNA ligase (NEB) overnight at 16°C. RNA was again phenol/chloroform extracted and precipitated with ethanol. Resulting RNA was reverse transcribed using SuperScript III reverse transcriptase (ThermoFisher Scientific) and cDNA was amplified using primers complementary to linkers and GoTaq polymerase for 25 cycles. CLIP was performed in three biological replicates. CLIP libraries were sequenced on Illumina HiSeq 2000 machine at the IMAGIF sequencing platform (Centre de Recherche de Gif – www.imagif.cnrs.fr). The input libraries used for normalization of CLIP signal were sequenced by NGS Facility at Campus Science Support Facilities GmbH (CSF), member of Vienna Biocenter (VBC), Austria.
